# Lectures

**Published:** 1844-07-13

**Authors:** Benjamin Collins Brodie

**Affiliations:** Consulting-Surgeon of the Hospital


### LECTURES

*Delivered in the Theatre of St. George's Hospital, in the session* 1843–44,

BY SIR BENJAMIN COLLINS BRODIE,

Consulting-Surgeon of the Hospital.

*Paraplegia affecting the upper extremities; its comparative rarity. Paralysis from caries of the spine. Diagnosis, and resulting differences of treatment. Paralysis from inflammation of the spinal cord. Influence of mercury and zinc. Hysteric paralysis. Palsy of particular nerves. Other kinds of partial paralysis.*

GENTLEMEN,—In my last lecture I described a class of paraplegic cases in many of which the paralysis affects the lower limbs first, then creeps upwards and attacks the upper limbs, the brain ultimately becoming affected. It is not, however, a matter of course that the paraplegia should begin in the lower limbs; it may commence in the upper limbs. It may be the result of disease affecting the upper portion of the spinal cord, that disease being either inflammatory or chronic—an alteration of structure, in fact; there being the same differences here as when paralysis affects the lower parts of the spinal cord.

There is on the table a preparation taken from the body of a gentleman whose case I will mention. He was a young man of irregular habits, drinking a large quantity of wine, and a good deal exposed to wet and cold in hunting. From this exposure to cold and wet he had a severe pain in the neck, which was supposed to be of a rheumatic character. He neglected it, went hunting, and drank wine as usual. In spite of this neglect the pains subsided, and he thought that the disease was gone. But about three months afterwards he became paralytic in one arm, and then in the other. The muscles were not all paralysed, for with one hand he could take hold of the other, and lift it out of its place; but after a time the arms became completely paralytic. He now came to London and placed himself under my care. There was tenderness of the neck, there appeared to be some enlargement of its posterior part, and by and by one lower limb became paralytic, and then the other. He subsequently became comatose, lay in that state some days, and then died. On examining the body after death we found the original disease to be that which you now see on the table. A tumour was inside of the theca vertebralis, but outside of the dura mater. There was a deposit of lymph, of considerable thickness, which had become organised, extending from the great occipital foramen down to about the fourth cervical vertebra, but it was not quite sufficient to press on the cervical portion of the spinal cord. Outside of the spine there was a quantity of coagulated lymph—a large mass along part of the bodies and sides of the vertebræ, and this communicated with lymph inside by processes of lymph extending through the openings by which the nerves passed out to form the cervical plexus. The immediate cause of death was effusion of fluid into the ventricles of the brain, that circumstance occurring in this case which I mentioned in the last lecture. The malady went on till the ventricles were attacked, and then the fatal disease was superadded to the original affection. There was no disease in any part of the spine below that I have mentioned.

In this case the upper limbs became paralytic first, and the lower afterwards, and that is the usual course where there is disease affecting the upper portion of the spinal cord. It is the case in disease of the vertebræ, as I shall mention presently.

A lady came to London some years ago to consult Sir H. Halford, and myself. She had become paralytic in the upper limbs, but that was all. She could walk about and do everything but use her upper limbs: and in these one muscle had given away after another till the paralysis was complete. She then began to experience considerable difficulty in swallowing, showing that the disease was not under the control of medical treatment, and we advised her to return to the country. She went, and there she died. I am not certain whether or not she became paralytic in her lower limbs; but her surgeon in the country examined the body after death and sent the result. The disease was confined to the cervical portion of the spinal cord, and from ramollissement similar to that which I described as taking place in the lower part of the cord, it was reduced to the consistency of cream.

Cases of paraplegia affecting the upper are not nearly so common as those affecting the lower limbs; but we see them every now and then. The opportunities of post-mortem examination, of course, are rare, but I have conducted two, and from these I should conclude that the seat of disease is generally to be found in the cervical portion of the spinal cord.

Caries of the spine produces paralysis of the parts below, as you are well aware, and so far there is a resemblance between the symptoms produced by caries of the spine and those diseases of the spinal cord to which I have adverted in this and the preceding lecture. Owing to this similarity between the symptoms of the two diseases, cases of paraplegia are continually supposed to be cases of diseased spine. This, however, is a great error, because the treatment proper in one case is quit a improper in the other. Where there is caries, it is necessary that the patient should remain a year or two in a recumbent posture, but that is not requisite in cases of disease of the spinal cord, and probably is sometimes injurious. In many cases of caries it is right to make caustic issues, apply setons to the back, and adopt counter-irritation; but, where there is disease of the spinal marrow, if these remedies are not useless, yet they torment the patient, make a great demand on his bodily powers, and besides exhausting his strength are sometimes absolutely injurious. Over and over again have I seen cases of paraplegia depending on disease of the spinal cord treated with caustic issues, seton, and blisters, but without being productive of the smallest benefit; on the contrary, they are generally prejudical, independently of which they make the patient miserable.

But how are we to distinguish cases of caries of the vertebræ from cases of paraplegia depending on disease of the spinal marrow? In the former there is generally pain in that part of the spine that is affected. There is one kind of caries, which I call rheumatic caries of the spine, in which the pain is very severe, and in which pain is produced by percussion on the spine; even in cases of scrofulous caries there is generally some pain in the part affected, and some pain on percussion, but it is not constant; and there are many cases of scrofulous caries in which this diagnostic symptom (pain) is absolutely wanting. This circumstance will help you in the diagnosis to a certain extent, but it is not of itself sufficient. If there be great pain in one part of the spine, and pain on percussion, you may be pretty sure that it is not disease of the spinal cord. I speak of pain that is indubitable, not imaginary pain. It is easy so to squeeze the processes of the vertebræ that the patient says it gives him pain. Still the absence of pain does not prove that the disease is not in the vertebræ, because in cases of scrofulous disease sometimes there is no pain. Paralysis, however, in cases of disease of the vertebræ, does not take place at an early period; it rarely occurs before there is angular curvature of the spine, and sometimes curvature to a considerable extent. That is a very important diagnostic mark. In cases of disease of the spine there is generally cramp in the lower limbs, and the posture of the patient is of a peculiar nature. The flexor muscles generally act, draw up the thighs and bend the legs, and you will find the patient getting into that position, with his knees drawn up towards the chest. By combining these diagnostic marks with each other you may generally make out whether the disease is within the theca vertebralis or external to it.

I now come to make some observations on the treatment of these cases, but it is rather difficult to lay down any clear rules for your guidance; that is, the treatment ought to differ according to the nature of the disease, but we have not yet sufficiently advanced in our knowledge of this complaint to be able to state positively whether the disease be of one kind or another. If the disease be an inflammatory affection of the membranes you may distinguish it tolerably well; but if it be of a chronic character it is difficult to discriminate between softening of the spinal marrow, tubercles in the spinal cord, and effusion of fluid into the theca vertebralis. I really am not able at present to tell you how to distinguish one of these diseases from the other in the living person, besides which the three may be combined together, or there may be one first, and the others may supervene.

However, let us suppose that there is a case, such as I have just described, of inflammation of the membranes of the spinal marrow. The patient comes to you with a severe attack of dreadful lumbago, and by and by he states that there is numbness in the legs, and then difficulty in moving them. In this case you may be pretty sure that there is inflammation of the membranes of the lower part of the cervical cord. How is that to be treated? In the first place take blood by cupping from the loins, and repeat it according to circumstances. Begin by purging the patient, clearing the bowels well out—a right plan to pursue in all cases of inflammatory disease. Then put the the patient under the influence of mercury, exhibit calomel and opium, and treat him as you would a patient labouring under pleuritis or iritis. If I am not much mistaken I have several times seen the disease stopped by the exhibition of mercury. I have known a patient labouring under numbness of the limbs aud incipient paralysis recover when the gums were made sore by mercury. But if you are called in at a late period, when inflammation has subsided, and the paralysis consequent on it remains, even then you cannot do better than put the patient under a course of mercury, though not such a course as you would employ in the beginning of the disease. You must not now exhibit two or three grains two or three times a day, but a mere alterative course —five grains of Plummer's pill, night and morning —the eight of a grain of bichloride of mercury twice a-day, in addition to which you may apply blisters to the lower part of the back.

The result will vary in different cases according to the time at which the treatment is commenced, or according to the intensity of the disease. In some cases you may obtain a perfect cure under the use of mercury; in others, an imperfect one. A gentleman riding in a second-class railroad-carriage was exposed to a draft of cold north-easterly wind from one to two hours. The next day there was pain in the neck, and two or three days afterwards his hands were benumbed. In the course of a week both his arms became paralytic, and then the lower limbs also. We put him under a course of mercury, and he partially recovered, so that he was able to walk about and write, but he was still paralytic to a certain extent.

The treatment of a chronic affection of the spinal cord producing paralysis, must be, to great extent, empirical, because you cannot make a certain diagnosis. Let me repeat what I have just now observed, that I have never seen any beneficial results arise from the use of counter-irritation; on the contrary, I have often seen it productive of mischief. Probably the bowels are very torpid,—they will require to be kept open, and it is very difficult to effect it. Sometimes very strong aperients are necessary for this purpose: but it is essential that they should be kept open, for the secretions of the digestive organs are very often exceedingiy disordered. The stools will be black, like tar, and the lodgement of the black secretion in the intestinal canal appears to be productive of great mischief to the system. Calomel and a black draught may be exhibited every now and then, but a patient cannot take them from day to day. Sometimes the comp. ext. colocynth will be sufficient, but simple purgatives often fail. The pills which I am about to mention, I have found to be convenient in cases of this kind. Two scruples and a half of comp. ext. colocynth; half a scruple of soap; one drop of croton oil. Let these be well rubbed up and carefully mixed, and divided into a dozen pills, one or two of which may be taken every night or every other night when wanted. These are excellent pills; they cause nothing like the inconveniences produced by large doses of croton oil, and are very efficient indeed. The disease is very probably quite incurable, and it does not matter what medicine you give the patient. But still every now and then the progress of the disease is stopped, and the patient gets very well again.

The treatment which I have found to be most successful, and under which I have seen the greatest benefit arise is, a grain of zinc made into a pill and given three times a day, and then a draught of twenty minims of tincture of cantharides to wash it down. If you dissolve the sulphate of zinc in the draught it makes it nauseous—you may as well give ink. After a time the sulphate of zinc may be increased, and if you please you may carry it up to five or six grains; but I do not advise you to do it, for if you increase it to a certain point it makes the patient sick, and you cannot induce him to take it afterwards. It is from the continued use of the zinc, and not from the exhibition of large quantities that benefit is to be derived. The zinc may be increased to a grain and a half, and the dose of tincture of cantharides may also be increased, but I do not advise you to go beyond what I have stated of the latter; for if you do it is very apt to irritate the urinary organs. The tincture of cantharides is a diuretic, and some have supposed that it does the most good when it acts as such; probably that may be the case, but it seems to be a stimulus to the nervous system also, I mentioned a case in the last lecture in which a gentleman became paralytic in the lower limbs from inflammation of the lower part of the spinal marrow, induced by a local disease arising from the tincture of cantharides swallowed by mistake. It is easy to suppose large doses of this agent my excite the vessels of the spinal cord so as to produce inflammation, and that very small doses may be a grateful stimulus to it, tending to restore its power in cases of paralysis. The best recoveries that I have seen have been under this treatment. Some patients have appeared to get very well again; in others the disease appears to have been suspended,—it has made no further progress. I see a gentleman every now and then who laboured under paraplegia, and in whom this treatment was employed. He is now able to walk about, though his limbs are still weak; he has been neither better nor worse for some years. In other cases I have thought that benefit has arisen from the long-continued use of very small doses of bichloride of mercury combined with tincture of cantharides. Small doses do not seem to act as mercury on the system. I apprehend it acts much in the same way as the sulphate of zinc. Exhibit the sixteenth of a grain of bichloride of mercury in a certain quantity of tincture of cantharides, in a draught three times daily, and such plan of treatment will sometimes be useful. But it is right to state that in a great number af cases of chronic paraplegia the disease is incurable. The disease, however, may go on for years before it ascends to the brain and destroys life.

I have described to you paraplegia affections occurring in cases of hysteria. These instances are not very uncommon, but paralysis arising from hysteria is very different from that originating from organic disease or pressure on the spinal cord. In hysteria the evil is not that the mucles do not obey the will, but the will is not exercised. It is a remarkable circumstance that a woman will be paralytic, think that she cannot use her limbs, and yet on something exciting or agitating her she can walk very well; and sometimes what is supposed to be paralysis in hysterical women is altogethet a cheat

A young lady was supposed to be paralytic in her lower limbs, but on some one going in to look at her they discovered her standing on a chair to reach down her bonnet. It is right that you should be aware of the tendency to practise deceit in all hysterical persons, and that you should make allowances for it; for it is a curious fact that some of those who are prone to deceive about their complaints turn out very well afterwards, and constitute some of the best members of society. One person will pretend to pass gravel which she has picked out of the earth; another will pretend to pass black urine which she made black by mixing ink with it; and another will pretend to be paralytic who is not paralytic at all. You should never expose these patients if you can avoid it, but try to get their attention directed to other things; for if you expose them, even to their own families, they will scarcely ever recover their character, whereas when the disposition to hysteria is removed many of them become excellent persons.

As this is not a systematic course of lectures, I am not particularly careful about the order in which I bring the subjects before you; and I shall conclude this lecture by adverting to some other cases of paralysis about which you will be consulted, of a different nature from those I have hitherto described. You will find a person paralytic on one side of the face, and nowhere else, and this may indicate some formidable disease, but that is not usually the case —there is no great mischief, and the patient gets well. The paralysis, if confined to one side of the face, does not excite any fear, as in the case of cerebral paralysis. It frequently arises from pressure or other injury affecting the portio dura. A person is exposed to a draft of cold air, and the next day one side of the face is paralysed, but it is unaccompanied with pain; the patient, however, becomes frightened, fancies that she is going to be paralytic, and her friends participate in the feelings. Let her be careful not to expose herself to the draft again, give her blue pill every night, an aperient every second or third day, let her live moderately, and in nine cases out of ten the muscles will begin to act, so that in two or three months she will be well. I cannot exacely say what is the pathology of such cases as I have just described. There is some deficiency in the nervous power; there may be inflammation of the neurilemma, or of the canal through which the nerve passes, but certainly there is no pain indicating its presence. There are, however, other cases in which there is clearly inflammation—inflammation of the petrous portion of the temporal bone. A gentleman was seized with terrible pain in the ear, it increased in severity, went to the head. became intolerable, keeping him awake at night, and making him almost delirious. One side of the face became paralytic, and he came to London just at that period. Dr. Chambers and myself were consulted on the case, and we concluded that there was inflammation of the petrous portion of the temporal bone extending from the tympanum. We cupped him again and again, put him under mercury, and made the gums sore. The pain then relaxed, the paralysis was gradually removed, and he got well. I saw him lately, and found him using one side of the face as well as the other. I believe that in these cases inflammation of the tympanum takes place first, and that it extends thence to the bones in the neighbourhood.

The treatment to be employed is that which I have just mentioned, and it almost invariably succeeds; namely, taking away blood, purging the patient, and making the gums sore with calomel and opium.

Partial paralytic affections may take place anywhere. A dropping of the eyelid—ptosis, from paralysis of the levator muscle—is not very uncommon. Occasionally it depends on something in the state of of the system, apparently without organic disease, causing an insufficient supply of nervous energy to the muscles. It may be relieved in some instances by a course of blue pill, occasional purgatives, and so on; but where it has existed for a long time, and these simple rules have failed in removing it, according to my experience it has originated in disease within the cranium, and you may expect to find deposit on the nerve there, or disease in that part of the brain from which the nerve arises A gentleman had tic douloureux of the face, he then had epileptic attacks and ptosis of one eyelid; the eyelid completely dropped. The body was examined after death, and we found the base of the brain—the cerebrum—in a state of ramollissement to a considerable extend. All that part of the brain from which the nerves had originated was in a state of softening, and this accounted at once for the epilepsy the tic douloureux, and the ptosis. Paralysis of the upper eyelid after an injury is not of serious consequence; it may arise from an extravasation of blood pressing upon the nerve, and that may be absorbed; but it is a very bad symptom when it follows inflammatory disease of the brain; for it is then generally the result of a deposit of lymph, or probably of matter, at the part whence the third pair of nerves has its origin.

It is not unusual to find partial paralytic affections in the lower limbs. A patient is exposed to cold, and then finds that he is unable to walk. On examination you discover that a part of the leg is numbed and some of the muscles, but not all, are paralytic. Put him on a course of blue pill, combine with it the use of some liniment, and he gets well. It is an affection of a nerve itself, not of nervous centres.

You will be consulted about children who are paralytic. There is a peculiar paralytic affection of the limbs that occurs in children who are very young. The child generally has a fit at the time which has terminated in water in the brain, and some time afterwards one or more limbs become paralytic, or one set of muscles in a limb and not in the other. In some cases the muscles at the back part of the leg become affected, the heel is drawn up, and the child grows up with contraction of the foot. It is necessary at some time or other to divide the tendon and relieve the contraction, Sometimes all the muscles of the lower limb become paralytic, and in other cases there is paralysis in one arm. I know a gentleman who, when he was an infant, had some affection of the brain, in consequence of which one arm became paralytic, and has continued so through life. Partial paralysis is often the cause of squinting; some of the muscles over the eyes become paralysed, and not the others.

I saw a child with a very singular paralysis of the following kind:—It seemed that the pharynx was paralysed or some of the muscles external to it which are necessary to deglutition, for it was with the greatest difficulty that he could swallow. It was evidently a paralytic affection which had come on suddenly without inflammatory symptoms. I never heard the result, but I suppose that the child must have died from starvation. It could scarcely take sufficient food to enable it to grow up. I really do not know what is the change produced in the brain in these cases. It does not appear to be of any great extent, and does not extend afterwards.

I need not state that every part of the body is represented in the brain. As the mandates of the will go from the brain to every muscle, so from every part of the body sensations are communicated to the brain, and injury to that part of the brain which belongs to a particular muscle may produce paralysis. The paralysis having once taken place it seems to go no further. It does not destroy life; but in most cases, being once established, it remains through life. The patient is never very well: he may, however, live to be old, and if you examine the brain you find nothing at all to account for the symptoms.

A paralysed limb does not grow like the other limb, and this is a source of great inconvenience in the lower limbs. As the child grows up, one leg is shorter than the other; some of the muscles may act and some not, but the whole of the limb suffers, and the patient is under the necessity of having a shoe with a thick sole to enable him to walk better.

If you are consulted on one of these cases in the first instance, I believe that you may do good by putting the patient under the influence of mercury. Even within the first two or three months it is well to try the effect of mercury on what I call, in order distinguish it, "infantile paralysis;" but after that, I do not think that it is worth while to have recourse to remedial measures. I have tried all sorts of remedies, and I have seen them resorted to by others, but I never saw any good arise from them. The best thing you can do for a patient growing up with paralysis in the lower limb is to consider whether any mechanical contrivance can be made use of to take the place of the paralysed muscles, and enable the child to walk about better then he would otherwise be able to move.—*London Lancet.*

